# Obstructive Jaundice as a Presenting Symptom of Acute Lymphocytic Leukemia in Adults: A Case Report

**DOI:** 10.7759/cureus.82004

**Published:** 2025-04-10

**Authors:** Sami M Al-Shehab, Tayseer K El Saheb

**Affiliations:** 1 Neurology, Hamad General Hospital, Doha, QAT; 2 Family Medicine, Primary Health Care Corporation, Doha, QAT; 3 Internal Medicine, Istiklal Hospital, Amman, JOR

**Keywords:** abdominal pain, acute lymphoblastic leukemia (all), adult, obstructive jaundice, rare association

## Abstract

We present a unique case of acute lymphoblastic leukemia in an adult. A 58-year-old male presented complaining of epigastric pain. He was initially diagnosed with obstructive jaundice, as indicated by an elevated ratio of direct bilirubin to total bilirubin levels. Bone marrow aspirate revealed hypercellular marrow with heavy infiltration of blast cells. The target cells were positive for CD20 by immunostaining. This finding is consistent with acute lymphoblastic leukemia B cell type. This case emphasizes that acute lymphoblastic leukemia can exhibit a wide range of symptoms and should be considered a differential diagnosis, especially when encountering atypical obstructive jaundice.

## Introduction

Acute leukemia is the uncontrolled proliferation of malignantly transformed hematopoietic stem cells [1], constituting the most common childhood cancer [2]. Conversely, acute lymphocytic leukemia denotes a malignancy of lymphocyte precursor cells [3]. Acute lymphoblastic leukemia constitutes approximately 12% of leukemia cases. Leukemic cells have the ability to migrate and invade different types of tissues [4] and are usually diagnosed via bone marrow aspirate. Moreover, acute lymphoblastic leukemia predominantly affects the pediatric age group, but carries a poorer prognosis if it presents in adults [5]. Acute lymphoblastic leukemia seldom manifests with abdominal pain and jaundice in adult patients. In this case report, we present a patient with acute lymphoblastic leukemia with right upper quadrant pain and jaundice.

## Case presentation

A 58-year-old male patient presented to the Emergency Department of Istiklal Hospital in Amman, Jordan, complaining of epigastric pain that was progressive, constant, and radiating to the right upper quadrant over the past two weeks. The pain intensified after consuming fatty food and was alleviated with vomiting. Concurrently, the patient displayed yellowish discoloration in both eyes, general weakness, nausea, and bilious vomiting on five separate occasions. Additionally, the patient reported dark urine but no change in stool color. The patient reported no changes in bowel habits, itching, recent weight loss, history of drug or alcohol abuse, or the use of pain medications.

Upon examination, the patient exhibited jaundiced sclera and skin, along with epigastric pain, tenderness in the right upper quadrant, and a positive Murphy’s sign. Consequently, the patient was diagnosed and admitted for obstructive jaundice, as indicated by an elevated ratio of direct bilirubin to total bilirubin levels, as seen in Table 1.

**Table 1:** Laboratory test results of the patient.

Test	Value	Normal range
Hemoglobin	12.3 g/dL	14–17 g/dL
White blood cell count	12.9 * 10 ^3/µL	4–10 * 10 ^3/µL
Neutrophils	17%	40–75%
Lymphocytes	76%	25–45%
Platelet count	62000/µL	140000–450000/µL
Creatinine	1.5 mg/dL	0.5–1.35 mg/dL
Urea	50 mg/dL	11–50 mg/dL
Uric acid	9.2 mg/dL	2.5–6 mg/dL
Sodium	142 mmol/dL	135–150 mmol/dL
Potassium	4.6 mmol/dL	3.5–5.3 mmol/dL
Total bilirubin	10.2 mg/dL	0.2–1.2 mg/dL
Direct bilirubin	6.2 mg/dL	0–0.3 mg/dL
Alanine aminotransferase	73 U/L	10–65 U/L
Aspartate aminotransferase	68 U/L	0–40 U/L
Gamma-glutamyl transferase	330 U/L	7–55 U/L
Alkaline phosphatase	179 U/L	40–136 U/L
Antinuclear acid antibody titer	<1:80	<1:80
Anti-smooth muscle antibody titer	<1:10	<1:10
Anti-mitochondrial antibody titer	<1:10	<1:10
C-reactive protein	18 mg/L	0–5 mg/L
Lactate dehydrogenase level	370 U/L	80–250 U/L
Phosphorus level	7.4 mg/dL	2.5–5 mg/dL
Hepatitis profile	Negative	Negative
Epstein-Barr virus	Negative	Negative
Cytomegalovirus	Negative	Negative
Prothrombin time	14.1 s	12–16 s
International normalization ratio	1.007	1
Partial thromboplastin time	32	25–35 s

**Figure 1:**
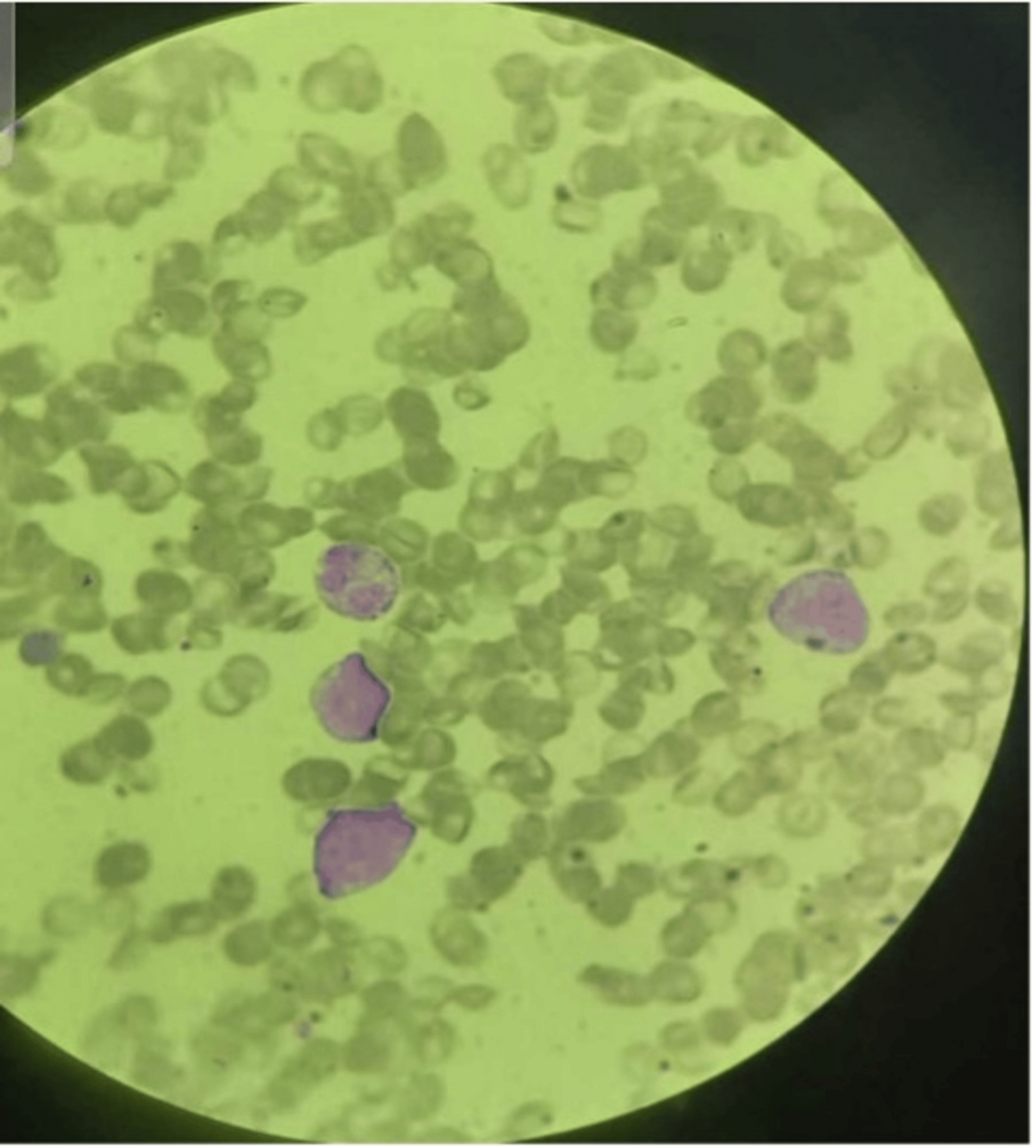
Blood smear exhibiting atypical lymphocytes.

Blood smear analysis revealed normocytic normochromic red blood cells with few macrocytes and polychromasia, anisocytosis, occasional normal red blood cells, normal white blood cells, few atypical lymphocytes, and marked thrombocytopenia (Figure 1).

**Figure 2:**
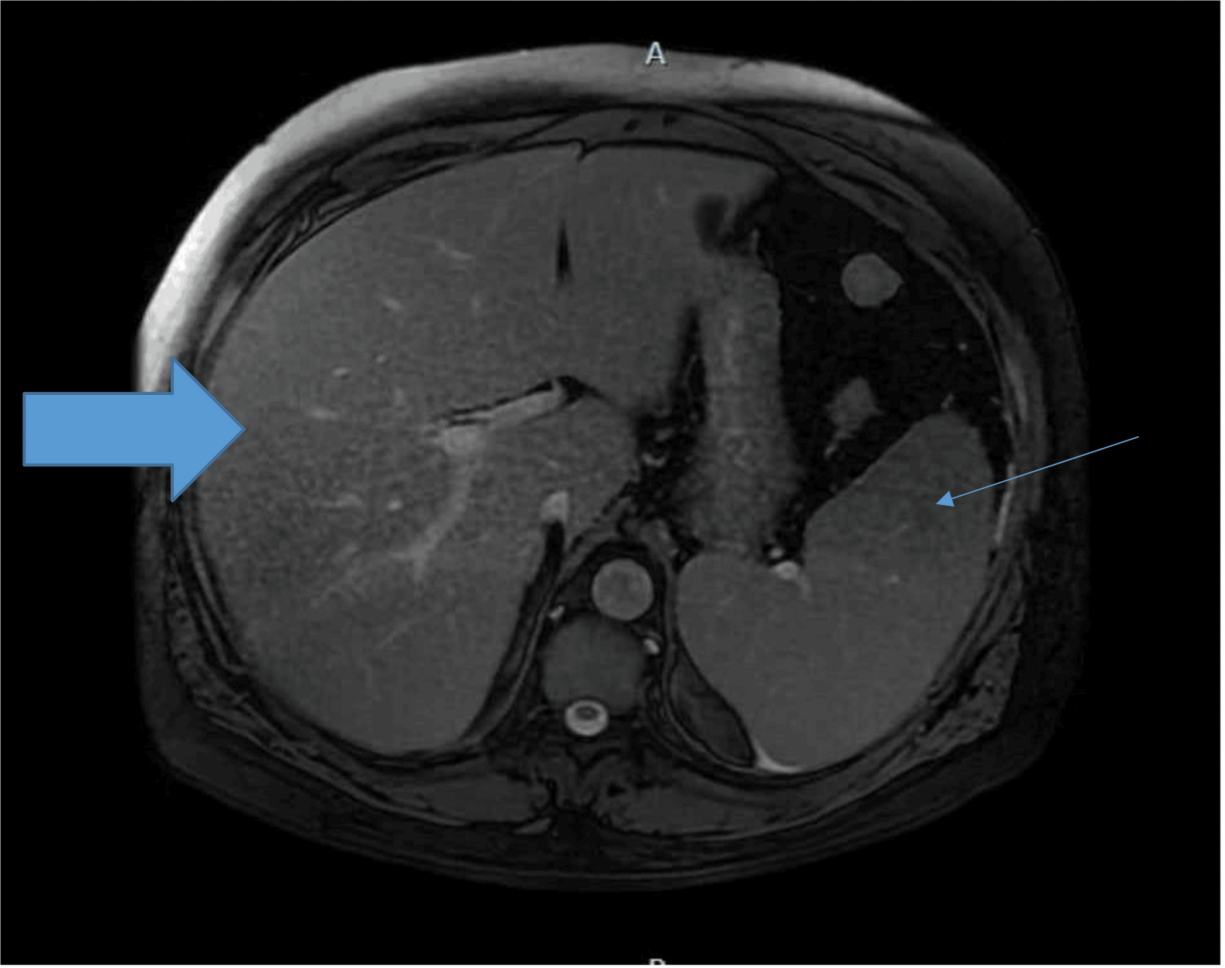
Magnetic resonance cholangiopancreatography revealing an enlarged liver and splenomegaly (thin arrow).

On magnetic resonance cholangiopancreatography, the biliary tree was not dilated, with a prominent left lobe of the liver and splenomegaly measuring approximately 14.7 cm (Figure 2).

The patient was initiated on ursodeoxycholic acid 150 mg orally every six hours (to promote secretion of bile acid), with intravenous fluid, and ceftriaxone 1 gm intravenously every 12 hours (due to suspected ascending cholangitis). Prednisolone 5 mg orally, two tablets every 12 hours, was added after magnetic resonance cholangiopancreatography revealed no biliary obstruction.

On day five following admission, the patient’s laboratory test findings revealed the following: white blood cell count of 0.7 * 10^3/µL, platelet count of 15 * 10^3 /µL, reticulocyte count of 1.4%, and total bilirubin level of 5 mg/dL (direct bilirubin level = 3.1 mg/dL).

**Figure 3:**
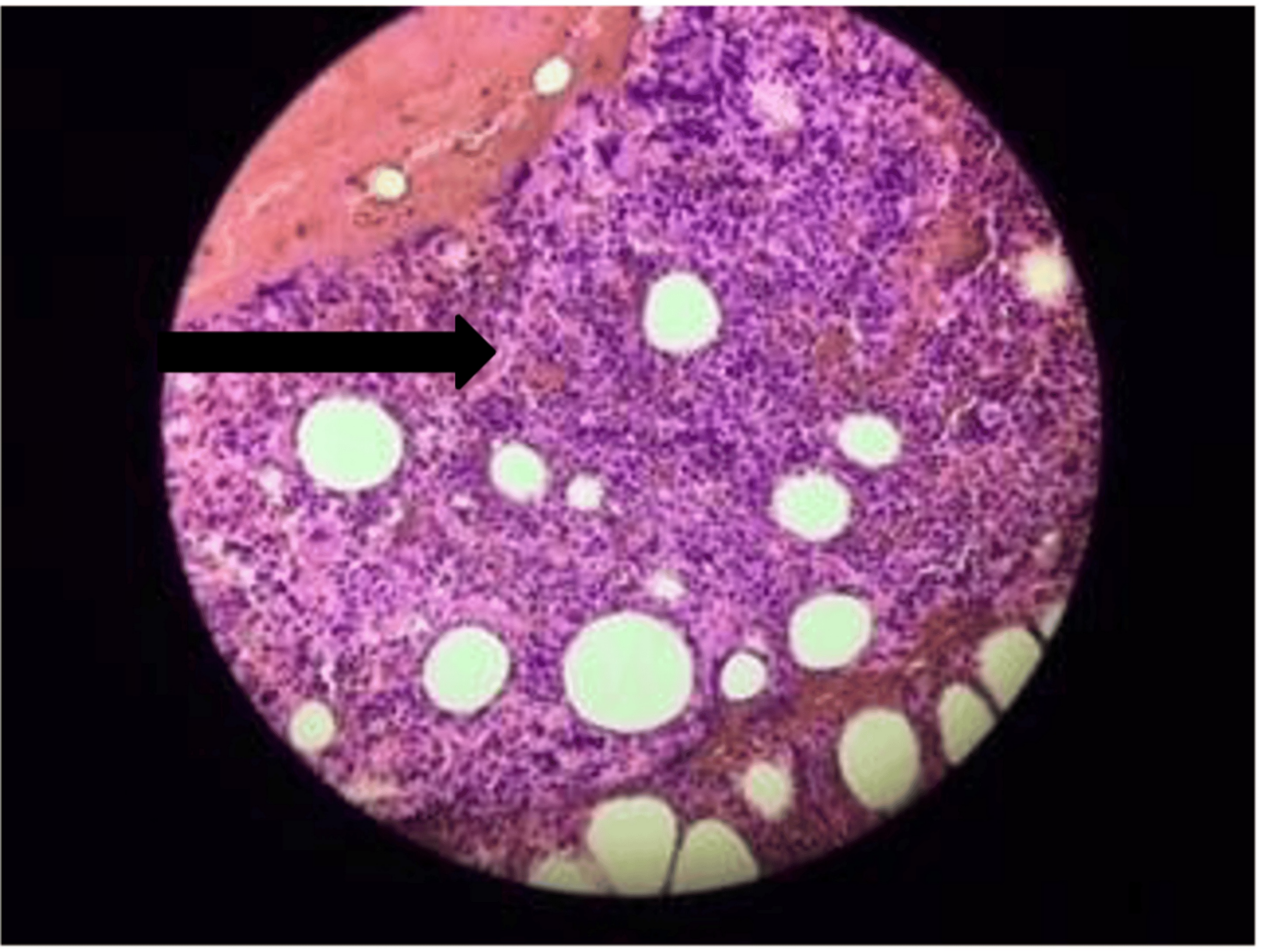
Hypercellular bone marrow with heavy infiltration of blast cells (black arrow).

Bone marrow aspiration was performed (indicated by the massively reduced white blood cell and platelet counts) on the same day. Analysis revealed hypercellular marrow with heavy infiltration of blast cells (Figure 3). Further, the target cells were positive for CD20 and negative for CD117, CD3, and myeloperoxidase by immunostaining. This finding is consistent with that of acute lymphoblastic leukemia B cell type.

Repeated blood smears were performed and revealed severe leukopenia, no blast cells, and severe thrombocytopenia. The patient was then initiated on chemotherapy and went into complete remission. He received the hyper-CVAD protocol, beginning with induction course A, including cyclophosphamide 300 mg/m² IV over two hours every 12 hours for six doses on days one to three with mesna uroprotection, vincristine 2 mg IV on days four and 11, doxorubicin 50 mg/m² IV on day four, and dexamethasone 40 mg orally (PO) on days one to four and 11 to 14, alternating every three to four weeks with consolidation course B, which comprised methotrexate 200 mg/m² IV over two hours, followed by 800 mg/m² IV over 22 hours on day one with leucovorin rescue, and cytarabine 3 g/m² IV over two hours every 12 hours for four doses on day two (reduced to 1 g/m² for patients >60 years), plus methylprednisolone 50 mg IV every 12 hours on days one to three for CNS prophylaxis. Maintenance therapy was planned post‑remission with 6‑mercaptopurine 75 mg/m² PO daily, methotrexate 20 mg/m² PO weekly, and monthly vincristine 2 mg IV plus prednisone 40 mg/m² PO on days one to five every three months, with each cycle administered at 21‑day intervals and close monitoring of hematologic and organ function.

## Discussion

To the best of our knowledge, we have encountered only two similar cases of acute lymphoblastic leukemia in patients presenting with right upper quadrant abdominal pain and jaundice similar to obstructive jaundice. In the first case, a 43-year-old male presented with epigastric pain and jaundice and was diagnosed with acute lymphoblastic leukemia [6]. The second case involved a 44-year-old male who presented with right upper quadrant abdominal pain and obstructive jaundice [7]. In both instances, the treatment approach consisted of standardized chemotherapy for acute lymphoblastic leukemia, supplemented by a short course of prednisone before initiating chemotherapy. There was a substantial reduction in bilirubin levels and alleviation of abdominal pain.

The mechanism behind the jaundice and abdominal pain is thought to be due to hepatic sinusoidal infiltration of leukemic cells [8]. The diagnosis is usually confirmed by bone marrow biopsy findings, revealing heavy infiltration of blast cells [7].

In conclusion, acute lymphoblastic leukemia can manifest in various ways, none of which are specific. Acute lymphoblastic leukemia should be suspected and included in the list of differential diagnoses when any abnormalities in complete blood count results are observed.

## Conclusions

Acute lymphoblastic leukemia in adults can present with atypical symptoms such as obstructive jaundice and right upper quadrant pain, mimicking hepatobiliary pathology. In any adult patient with unexplained cholestatic liver enzyme elevations or obstructive jaundice, especially when routine imaging is non‐revealing, peripheral blood counts should be scrutinized for blasts or cytopenias. Prompt bone marrow evaluation remains the gold standard for diagnosis. Early recognition and initiation of standard acute lymphoblastic leukemia chemotherapy protocols, often preceded by a brief course of corticosteroids, can rapidly reverse hepatic infiltration, normalize bilirubin levels, and induce remission.
